# AMXRA Guidelines on Extended Reality (XR) and Children: Considerations for Safe and Effective Application Development and Use

**DOI:** 10.1089/jmedxr.2024.0054

**Published:** 2025-04-14

**Authors:** Asher Marks, Veronica Weser, Thomas Jon Caruso, Susan Persky, Abraham Homer, Jeffrey Gold, Erin Williamson, Kimberly Hieftje

**Affiliations:** ^1^ Yale School of Medicine, Department of Pediatrics; The Yale Center for Immersive Technologies in Pediatrics, New Haven, CT, USA.; ^2^ Stanford University School of Medicine, Division of Pediatric Anesthesiology, Palo Alto, CA, USA.; ^3^ Social and Behavioral Research Branch, National Human Genome Research Institute, Bethesda, MD, USA.; ^4^ Children’s Hospital Colorado, Aurora, CO, USA.; ^5^ Keck School of Medicine, University of Southern California, Departments of Anesthesiology, Pediatrics, and Psychiatry & Behavioral Sciences; The Saban Research Institute at Children’s Hospital Los Angeles, Department of Anesthesiology Critical Care Medicine, The Biobehavioral Pain Lab, Los Angeles, CA, USA.; ^6^ Love146, New Haven, CT, USA.

**Keywords:** child development, extended reality, health, safety, virtual reality

## Abstract

The extended reality (XR) revolution has ushered in the adoption of head-mounted displays (HMDs), including among children. While well-studied clinical uses of XR hold promise for positive outcomes in pain management, behavioral change, and social connection, unsupervised use and improper implementation of immersive technologies in young populations raise significant concerns. Current age limit recommendations are based primarily on privacy laws and fail to address the potential physical, cognitive, emotional, and other psychosocial implications of XR for children. Commercially available HMDs are ill-suited for young users, leading to discomfort and suboptimal immersive experiences. Further, XR experiences challenge young children’s ability to differentiate reality from fantasy, resulting in safety hazards and the potential for confabulated memories. Concerns extend to immersive social experiences, where the impact on mental health and safety is not yet fully understood. While acknowledging the risks, embracing XR technologies with thoughtful consideration is necessary to maximize their potential benefits for all youth, especially those facing adversity and isolation. Balancing accessibility and safety is essential as current and future XR technologies become integrated into children’s daily lives. This article represents AMXRA’s official position on the responsible use of XR in children and will address the three major components of childhood development-physical, cognitive, and psychosocial—as they relate to the use and development of immersive technologies, in homes, schools, and clinics.

## Introduction

Extended reality (XR) is an overarching term that encapsulates augmented reality (AR), virtual reality (VR), and mixed reality (MR) applications that most often use head-mounted displays (HMDs) to display digital content to users.^1^ HMDs are devices worn on the head in the form of glasses, helmets, or goggles, that position small screens in front of the user’s eyes to create a three-dimensional (3D) effect by tracking head movements to create an immersive digital or digital/physical world hybrid. HMDs usually incorporate headphones or speakers for spatial audio, adding to the overall experience. This effect makes the use of XR in younger populations potentially problematic as technology users find themselves completely immersed, resulting in digital environments that can be difficult to discern from reality.^2^ At this time, these technologies are frequently viewed from an entertainment perspective, but current data on the use of immersive technologies with children comes from clinical and educational usage. Among other uses, XR use in children has shown great promise in acute and chronic pain management,^3–5^ treatment of anxiety-related disorders,^6,7^ social and emotional skill development for children with autism,^8,9^ improvement in attention in children with ADHD,^10,11^ and increased balance and coordination of children with cerebral palsy.^12,13^

Beyond the use of XR to improve health and psychosocial outcomes in children in supervised settings, adoption rates of HMDs in the general population have exponentially increased over the past five years, rivaling peak adoption rates of technologies such as color TV, personal computers, and mobile phones.^14^ A 2021 Common Sense Media report found that 17% of 8–18-year-olds in the United States have a VR headset in the home with VR use most commonly reported among boys and children from lower-income households.^15^

Current recommendations for most commercial HMDs specify that users must be above 12 years to safely use them, though Meta, Inc. (Menlo Park, CA, USA) implemented parentally controlled accounts for children ages 10–12 at the end of 2023.^16^ Current recommendations are primarily based on The Children’s Online Privacy Protection Act,^17^ which was enacted in 1998 and is intended to prevent online platforms from collecting the personal data of children under the age of 13 for advertisement targeting and tracking. These recommendations do not consider the physical, cognitive, and psychosocial development of children specific to technologies such as XR. Currently, the broad impact of XR with HMDs on children is theoretical and largely unstudied, especially outside the clinical or laboratory space.

As XR and HMDs become increasingly prevalent and accessible among children, it is imperative to deepen our understanding of how these technologies intersect with their physical, cognitive, and psychosocial development. Leveraging our current understanding of child development and existing, though limited, research on children’s use of XR, we aim to provide practical guidelines for the responsible and safe use of XR technology and HMDs within this vulnerable population based on an understanding of child development, current literature, and expert opinion. The following recommendations represents AMXRA’s official position on the responsible use of XR in children.

## Physical Development of Children and XR

While children are eager to interact with new technologies, their development in several specific physical areas complicates their participation in XR experiences. Areas of most concern include fine motor skills, postural control and core strength, and balance. Commercially available HMDs are designed for adults: specifications that inform the design of commercial HMDs include the size and weight of the headset, the average adult’s interpupillary distance, and the design of corresponding hand controls.

**FIG. 1.**
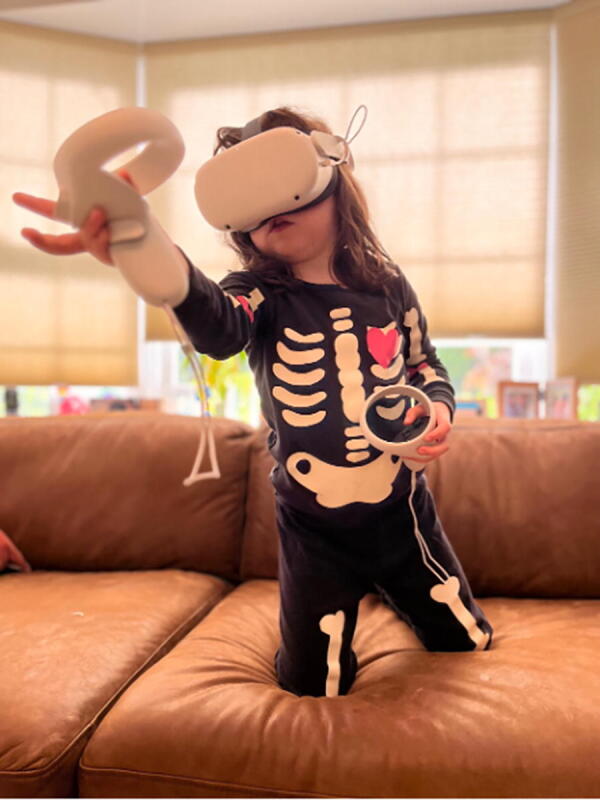
A 7-year-old female engaged in a lively virtual reality session of “Beat Saber.” She is on a couch to allow her to get to the height of her avatar and the headset is sitting low on her face, covering her nose completely. She is also having difficulty reaching the appropriate hand controls on the controller and is struggling with maintaining balance. She is at risk of falling off the couch.

Underdeveloped fine motor skills, coordination, and small heads and hands in children may result in difficulty navigating the virtual and augmented worlds they encounter when wearing an HMD (Fig. 1). Though becoming less common, many XR encounters require the user to reach a variety of sensors and buttons on handheld controllers. If a child’s fingers simply cannot reach around the controls appropriately or have not developed the fine motor skills to navigate the controllers, the experience may become frustratingly unusable, and hand cramping and discomfort may ensue. As XR applications increase the adoption of controller-free experiences, and rely on hand tracking, these newer approaches may pose additional challenges such as tracking inaccuracies, misinterpretation, and fatigue, as the technology was developed using adult hands. For instance, newer headsets such as the Apple Vision Pro utilize hand gestures and eye tracking to navigate applications without any controllers at all. This integration of gesture-based controls can create more flexibility in how users interact with immersive experiences; however, this may also introduce limitations to children with underdeveloped fine motor skills or mobility restrictions.

Based on the combined experiences of the authors, HMDs are often too big and too heavy to be comfortably used by younger children. Using these larger headsets may cause headaches and neck strain after being used for even short periods. The size discrepancy between the child and the headset also alters the focal “sweet spot,” or the area of the lens that creates a clear image when directly viewed. In HMDs, particularly those that lack “pancake lenses” (compact, lightweight lenses that are used in VR headsets to reduce device size and improve image clarity by folding light paths through polarization and reflections), the focal point is very specific. When this focal point is not precisely set, the image becomes blurry, further contributing to headaches, eye strain, and a suboptimal immersive effect. These concerns are magnified when considering the pupillary distance in children as compared to adults.^18^ While some headsets allow the pupillary distance to be adjusted from adult levels (54–74 mm) down to pediatric levels (43–58 mm), most do not.

While a significant amount of attention has been given to the concern over the use of HMDs on a child’s developing vision, the American Academy of Ophthalmology recently published a statement that “Although there are no long-term studies, ophthalmologists agree there is no reason to be concerned that VR headsets will damage eye development, health or function.”^19^ Several studies have explored using VR for the assessment of amblyopia^20^ and strabismus,^21^ with some even investigating the use of VR for myopia treatment.^22^ There is currently a Food and Drug Administration-cleared VR-based amblyopia treatment for children on the market for children as young as 4 years of age.^19^

Vergence accommodation conflict (VAC), however, must be considered in children who are still developing their vision. VAC occurs when vergence or the inward/outward rotation of the eyes to converge on an object, is not in sync with accommodation or the contraction of the lens to focus. This becomes an issue in HMDs as the vergence system responds to the virtual distance of objects, adjusting as if they are near or far. The accommodation system, however, is fixed to the physical distance of the VR display, which is typically just a few inches away from the user’s eyes. VAC can lead to eye strain and headaches, making the VR experience uncomfortable, especially over an extended period. The development of permanent vergence accommodation pathologies after extended use of HMDs has not been well studied, however, there is preliminary evidence that extended use of digital devices during the COVID pandemic did cause acute visual issues in children, suggesting further research is needed on HMDs.^23^

Children may also be subject to the experience of uncomfortable symptoms experienced by XR users, including dizziness, disorientation, eye strain, headache, pallor, sweating, increased salivation, fatigue, drowsiness, stomach awareness, nausea, and sometimes vomiting. Often called cybersickness, simulator sickness, or simply motion sickness, these symptoms occur in users of all ages. Discomfort varies in severity and frequency depending on the XR experience, but is especially common during the use of lower-quality immersive experiences (those with tracking latency or lag) or when there is a high degree of mismatch between the user’s virtual and physical movements,^24^ such as when a user moves through a virtual environment with a joystick. Although a recent systematic review examining the safety of VR use by children found only limited data suggesting mild symptoms,^25^ these outcomes are dependent largely on features of the hardware and software used in contributing studies. In addition, it is well accepted that the onset of motion sickness susceptibility begins around age 6 or 7 years,^26^ and peaks at around age 9 or 10 years,^27,28^ suggesting that this age group might be among the most vulnerable to this unpleasant XR-associated phenomena. Beginning around age 12 or 13, there is a decline in the susceptibility of motion sickness that is usually attributed to habituation.^29,30^ However, some adult users^26^ experience motion sickness-like symptoms when using XR technology, even when spared from motion sickness in canonical situations like boat and car rides.

The most accepted explanation of motion sickness is that the negative symptoms are brought on because of the sensory conflict or sensory mismatch between the actual and the expected patterns of vestibular, visual, and kinesthetic inputs. VR is particularly conducive to this type of mismatch because the HMD fully replaces one’s perception of the external world. Thus, although the vestibular and somatosensory systems may signal that the child is (relatively) still, the visual system may receive input from the VR display that the child is running or jumping through a virtual environment using buttons or joystick presses on the device’s handheld controllers.

The leading alternative explanation for motion sickness is the theory of postural instability,^31,32^ which posits that motion sickness and by extension, cybersickness, and simulator sickness, occur with a loss of postural control (i.e., the ability to keep a stable, balanced body position). Many studies of adult users of XR technology have shown that an individual’s spontaneous postural instability can predict their likelihood of experiencing motion sickness during HMD usage,^33–36^ and that the onset of unpleasant symptoms is preceded by an increase in postural instability.^37^ Because children and adolescents must continuously recalibrate their proprioceptive systems to account for body growth, multisensory integration strategies do not reach adult levels until young adulthood.^38,39^

### XR development and use considerations in the context of physical development

Children’s physical development presents unique challenges to their engagement with XR. Problems related to the physical fit and suitability of HMDs, the coordination needed to interact effectively with XR environments, cybersickness, and potential effects on vision must be considered when designing XR applications and HMDs.

HMDs would benefit from a range of fits and configurations that would make their use more accessible to smaller heads and weaker necks. While weight and overall size are a consideration, so too, should be the option to avoid complete immersion and separation from the surrounding environment. This can be accomplished by focusing child-facing interventions on MR approaches—eliminating completely immersive environments when viable. With this approach, however, we must also consider several unknowns. Most notably, the effects of MR on a child’s ability to differentiate between real and virtual experiences and subsequently real-world versus digital-world memories. An alternative or modified approach to full immersion includes the option of headsets with an open periphery, allowing outside light and peripheral vision to remain intact. An example of this is the Meta Quest Pro^TM^, which keeps the edges of the HMD-face interface open, allowing peripheral light and surroundings in. There are similar third-party accessories to allow for modification of various off-the-shelf headsets to do the same.

Additional hardware considerations include smaller hand controllers designed for children, or control systems that avoid hand controllers completely, including hand, head, or eye tracking. Concerns regarding coordination and poor fine motor skills can be addressed through larger target areas (invisible boxes around an object that tell the software where a player can interact with the object) within experiences. For example, while a virtual object may be only 6 inches in size, creating an 8-inch target around it to account for a child’s less-developed motor system may be helpful. As HMDs and immersive software continue to develop, there will be a much greater consideration for flexible control schemes and adaptive hardware solutions.

Finally, given the developmental course of postural control and the known associations between postural instability and motion sickness symptoms, the susceptibility of children to transient imbalances following the usage of XR technology is worthy of investigation. Thus far, research investigating the aftereffects of children’s VR usage has shown only negligible passing balance changes.^40,41^ Taken together, the best ways to reduce the incidence of cybersickness for users of all ages is to reduce discrepancies between virtual and physical body movement by avoiding experiences in which virtual distances are traveled using joysticks or other non-body-based navigation schemes and to encourage users to maintain a stable, seated posture throughout the experience.

## Cognitive Development Of Children and XR

Cognitive development is the progressive and continuous growth of perception, memory, imagination, conception, judgment, and reason.^42^ Based on various theories and frameworks that seek to explain cognitive development,^43–46^ younger children (ages 2–6 years) are often characterized by concrete thinking, egocentrism, constrained perspective-taking, poor spatial awareness, and limited working memory capacity. Older, school-aged children (ages 7–12 years), develop the ability for logical thought within concrete contexts, have increased spatial awareness, have an enhanced working memory capacity, and can comprehend others’ viewpoints and feelings. Starting around 13 years, adolescents are capable of abstract reasoning,^47^ have a more complex self-concept and exploration of personal identity,^48^ large working memory capacity, and an enhanced understanding of complex social relationships and perspectives. While greater complexity in cognitive development is critical for adolescents as they work towards transitioning to adulthood, there is also an increased propensity for risk-taking and poor judgment due to the ongoing development of the prefrontal cortex during this period.^49^

The use of XR with children presents unique cognitive challenges. Because younger children are still developing spatial awareness, reasoning, and understanding, they may have difficulty understanding the intricacies and relationships of 3D objects in VR.^50^ Younger children also have difficulty filtering out distractions within VR, resulting in a reduction in attention and self-control while also creating high cognitive load demands.^51^ Because self-awareness and distraction influence an individual’s perception of presence, young children are likely to experience virtual presence differently than older children and adolescents.^50^ Younger children may also be unable to differentiate between real and virtual experiences, leading to confusion and false memory creations within VR, also known as confabulated memories or reality monitoring errors.^52^ For instance, a study published in 2009 found that children 6–7 years who watched their virtual self in VR swim with orca whales were confused if their memory was that of a virtual experience or real life.^53^ Likewise, younger children who are unable to differentiate between realities may experience a profound sense of immersive presence and become highly influenced by both positive and negative content. Younger children’s inability to resist distractions and exert self-control within VR environments could create highly intense, over-stimulating experiences leading to fear, confusion, and discomfort.

Older children (ages 7–12) are more able to distinguish between the virtual world and the real world, and this understanding continues to develop into adolescence. They are often able to fully engage with complex narratives and problem-solving tasks involving spatial awareness within VR, allowing them to benefit from educational or therapeutic applications. More immersive and nuanced VR experiences may be better suited for older children who can engage in abstract thinking and complex problem-solving, have greater self-awareness, and can filter out distractions. While the appropriateness of content and the potential psychological effects of extended VR use are still concerns for this age group, they can be managed with proper supervision and consideration.

A further concern regarding the use of HMDs in pediatric populations is reduced inhibitory control in young children. The distinction between virtual and real worlds becomes blurred, and physical environmental awareness is low, creating physical hazards outside of the HMD. Despite the use of software-based “guardians” triggering a digital barrier or a headset’s passthrough to enable viewing of the user’s surroundings, this lack of inhibition and differentiation between reality and fantasy can result in spontaneous runs, kicks, or punches into walls, furniture, pets, and people even before a guardian can inhibit this behavior. As enthusiasm regarding the potential benefits of XR technologies in the pediatric population continues to grow, we must consider hardware that is specifically designed for a child’s unique physical attributes and software designed to ensure they remain safely within virtual “guardians.”

### XR development and use considerations in the context of cognitive development

It is essential to consider the cognitive abilities of children when designing and choosing appropriate XR content. Younger children may struggle with 3D navigation, distinguishing real from virtual, and may be more susceptible to the influences of virtual content. Thus, the design of XR applications for younger children should aim to limit cognitive load and multi-sensory overload that could lead to an overwhelming XR experience. Experiences designed for children should be clearly distinguishable from reality to prevent confusion or the creation of false memories. Some proposed approaches include avoiding photo-realistic graphics and incorporating reminders in the form of persistent visual idiosyncrasies that the experience is fabricated. This may be accomplished through such variances as persistent and specific borders on virtual objects, changes in color saturation, or a limited palette for virtual objects.

Older children can engage more effectively with complex XR content, especially ones that require spatial thinking and reasoning, but content suitability, psychological effects, and online safety are important concerns. Responsible adults should closely supervise the XR content and duration of use for children of different ages. For instance, some HMDs, such as the Meta Quest devices, allow the player to cast their screen to a web-based device such as a computer or phone which can readily be used for monitoring.

This supervision can be approached in several ways and can be divided into active and passive approaches. An active approach would include an adult being present during the use of the headset with the content of the experience being continuously streamed to a nearby screen that an adult can monitor. Such approaches are already available as commercial solutions and built into most HMD operating systems. In addition, these approaches are frequently used in pediatric hospitals when the users are young and/or undergoing procedures.^54–56^ A passive approach to supervision would be through ensuring that headsets are set in kiosk mode (allowing a device to run only specified applications and settings) or having only acceptable applications loaded onto the headset without access to the internet.

Further, modifications to an HMD’s guardian system, that is, the system that triggers a digital marker or the headset’s passthrough when the user reaches the edges of the play area, may be helpful when dealing with a child’s lessened inhibitory control.

When children are using the system, we recommend the guardian be set intentionally smaller to trigger passthrough earlier, or even more conservatively, aim for a seated experience. In addition, newer headsets have added sensors and tracking systems such as lidar (light detection and ranging), passthrough, and the ability to include and exclude real-world objects. With the addition of MR mechanics, as mentioned previously, further set-up factors should be considered based on the features and capabilities of the hardware.

## Psychosocial Development in Children and XR

Psychosocial development in children refers to the growth of a child’s emotional and social capabilities,^57^ emphasizing the needs of the child and their interactions with their social environment. During childhood, social learning impacts a child’s psychosocial development through social interactions within a cultural context.^58^ Early childhood is a formative period for children to develop an understanding that others have thoughts, feelings, and perspectives that differ from their own. This revelation is essential for the development of empathy^59^ and personal identity.^60^ As children grow, they learn to establish and maintain peer relationships, lessons that are required to later develop skills around sharing, cooperation, and conflict management.^61^ During adolescence, peer relationships become highly influential, even more so than family relationships. Peers become an important source for emotional support and belonging,^62^ social skill development,^63^ and identity formation.^64^ For children and adolescents, peer interactions influence decisions around risk-taking behaviors. While positive peer interactions can lead to prosocial behaviors and healthy decision-making, negative peer influence can lead to risky behaviors and harmful choices.^62^

Because of its immersive nature, XR can provide children with opportunities for interactive and experiential learning, especially within the context of social learning.^8^ The technology allows for the creation of virtual situations that potentially promote empathy through embodiment and perspective-taking,^65^ such as experiencing the marginalization of a specific group. It is important to note, however, that some studies have demonstrated backfire effects resulting in decreased empathy in certain VR contexts, highlighting the importance of content monitoring.^41,66^ Importantly, XR applications also provide opportunities for older children and adolescents to practice peer refusal and healthy decision-making while exploring the consequences of risky behaviors within a safe space.^67^

Social interactions in VR allow children and adolescents to interact with others within a shared virtual space, which can provide a platform for social learning, shared experiences, and skill development. The use of unvetted, unsupervised social experiences within XR for children, however, may cause harm in the form of privacy breaches, negative psychosocial effects from intense XR experiences, cyberbullying, and online predators.^68^ VR, by design, is an immersive and embodied experience. The user is meant to emotionally and physically feel the connections made in the virtual world. As such, early research suggests that in some cases trauma experienced in VR results in physiological responses similar to those that occur during real-world physical and emotional trauma.^69^

### XR development and use considerations in the context of psychosocial development

XR offers opportunities for social learning and the support of emotional development. The immersive nature of XR amplifies both the positive and negative social interactions children may encounter. This can range from empathy building through embodiment in XR to practicing social interactions and communication skills in virtual environments.^66,70,71^ It can provide a sense of community and support, especially for marginalized groups like LGBTQ+ youth, but also increases exposure to harmful social interactions and potential trauma. As with physical and cognitive development, careful consideration should be given to the nature of these interactions and the content of the experiences.

Some technology companies are addressing these concerns in the form of virtual barriers to prevent people from getting within a certain defined personal space, and virtual “eject” buttons that bring the user to a safe space if they begin to feel threatened by other users. Children’s use of XR, however, should still be closely monitored, particularly in social VR experiences to minimize any form of bullying, inappropriate interactions, and unauthorized data collection or sharing. In addition to monitoring, it is critical to evaluate the rules, norms, and culture of social XR platforms before allowing access as many XR social spaces are broadly inappropriate for children. This approach may provide teachable moments by helping children understand how to navigate difficult digital social interactions.

Default settings are powerful tools for influencing choice.^72^ In general, humans tend to opt for the status quo, thus accepting the default outcome.^73^ In the case of technology, parents and other caregivers may assume that the default settings are enough to protect children. Unfortunately, this may not always be the case.^74^ It is therefore important that a trusted adult further ensure the protection of children by assisting in reviewing and setting privacy restrictions on their devices and platforms. This approach will help children learn about privacy concerns and how to best protect themselves when online.

While the above recommendations assume social human-to-human interactions, it is also critical to consider interactions with non-playable characters (NPCs)-both, those driven through standardized conversation trees and those driven by large language model artificial intelligence (AI). NPCs driven by conversation trees provide developers an opportunity to guide conversations toward the best outcomes. In the context of generative AI-driven NPCs, where conversational outcomes cannot be directly controlled by the content creators, it is important to implement strong guard rails for when these NPCs interact with children. In either case, the age and development of the intended audience should be considered in the creation of NPCs; these considerations may need to include assumptions about the inability of young children to differentiate a virtual human from an actual one.

## XR Development and Use Considerations Summary

While developmental milestones can be broadly placed within the context of specific age ranges, every child is different and develops at their own pace. Based on our understanding of children’s development, and to mitigate potential health risks, we recommend age-specific time limits per session for VR usage. Children aged 7–12 should limit sessions to 10–15 min at a time with frequent breaks to reduce eye strain, motion sickness, and the challenges of interacting in immersive environments where motor coordination and cognitive control are still maturing. Adolescents aged 13–17 years can extend session time to 20 min at a time, considering their increased ability to manage complex spatial tasks and maintain focus within VR, yet still require breaks to avoid prolonged physical and cognitive strain.^75^ These conservative guidelines align with the limited literature available and manufacturer recommendations that suggest cautious, time-limited exposure to VR for younger users (Table 1). As more research is conducted in this area and as XR technology advances recommendations and guidelines will need to change as well.^76^

**Table 1.** Recommendations and Guidelines for XR and HMD Use in Children

Age group	Recommended session time	Supervision recommendations	Content and development guidelines
Under 7 years	Not recommended outside clinical/research settings	Strict supervision is required, ideally in clinical or research settings only No social interaction features	Use non-immersive or partial immersion experiences to reduce confusion between real and virtual worlds Content should be simple and age-appropriate Content should avoid any elements that may be too immersive or overstimulating Avoid complex interactions and prolonged use Disabled data collection to protect privacy
7–12 years	10–15 min at a time Encourage regular breaks to mitigate physical strain	Close supervision with active adult guidance including possible screen casting for visibility Use conservative guardian boundaries and/or seated experiences	Pediatric-specific content, non-violent content Limited sensory engagement to avoid overload Limited or no social interaction features Disabled data collection to protect privacy
13–17 years	Up to 20 min at a time Encourage regular breaks to mitigate physical strain	Supervision recommended, especially in social VR environments Enable privacy and safety settings with conservative defaults Direct supervision and vetting for social interaction	Adolescent-specific content suitable for cognitive and psychosocial development level Content should promote positive social interactions Avoid overly intense VR experiences to reduce motion sickness Disabled data collection to protect privacy

HMD, head-mounted displays; VR, virtual reality; XR, extended reality.

## Conclusions

As the ubiquity of XR technologies and HMDs continues to grow, it is vital to understand their impacts on children’s development and create tailored safety guidelines. These guidelines should be mindful of each child’s unique pace of development and mitigate the risks associated with XR. Developers should design and adapt XR experiences to align with an understanding of children’s development, including considerations for physical fit, fine and gross motor coordination, cognitive load, content suitability, and social interactions. Similarly, adults working with children should ensure a balance between the immersive, and educational potential of these technologies and their potential adverse effects. There is a need for research in this field to expand our understanding of XR and children, especially as it relates to their physical, cognitive, and psychosocial development. We expect our recommended guidelines and considerations to change and expand as more research within this field becomes available.

While XR has significant potential to support and enhance children’s development, it must be used thoughtfully and responsibly. Its adoption should be guided by an understanding of children’s physical, cognitive, and psychosocial development. As new technologies develop, such as the use of integrated AI in XR experiences, improved and more easily available MR, and advanced haptics, future research should further investigate the long-term effects of XR use on children’s development to ensure this technology’s benefits outweigh its potential risks.

## Abbreviation Used

3D: three dimensional
AI: artificial intelligence
AR: augmented reality
HMD: head-mounted display
MR: mixed reality
NPC: non-playable character
VAC: vergence accommodation conflict
VR: virtual reality
XR: extended reality
